# Substance (mis)use among refugees as a matter of social ecology: insights into a multi-site rapid assessment in Germany

**DOI:** 10.1186/s13031-023-00499-9

**Published:** 2023-01-19

**Authors:** Laura Hertner, Panagiotis Stylianopoulos, Andreas Heinz, Ulrike Kluge, Ingo Schäfer, Simone Penka

**Affiliations:** 1 Department of Psychiatry and Neurosciences at the Charité Campus Mitte, Charité – Universitätsmedizin Berlin, Corporate Member of the Freie Universität Berlin and Humboldt-Universität Zu Berlin, Charitéplatz 1, 10117 Berlin, Germany; 2 Berlin Institute for Empirical Integration and Migration Research at the Humboldt Universität zu Berlin, Berlin, Germany; 3 Department of Psychiatry and Psychotherapy, University Medical Center Hamburg-Eppendorf, Hamburg, Germany; 4 Center for Interdisciplinary Addiction Research, University of Hamburg, Hamburg, Germany

**Keywords:** Alcohol, Drugs, Refugees, Social ecology, Refugee shelters, Work permit, Ecological model of refugee distress

## Abstract

**Background:**

Previous research concluded that substance (mis)use is increasing among forcibly displaced populations. Nevertheless, little research has been conducted within a social ecological framework aimed at identifying and understanding the factors affecting substance (mis)use embedded in the post-migration context in high-income countries. The present study aims to develop an understanding of the links and underlying mechanisms between refugees’ social ecological determinants and substance (mis)using behavior.

**Methods:**

Rapid assessments (RAs), including 108 semi-structured interviews and 10 focus group discussions with key persons from various professional, and personal backgrounds, were carried out in German urban and rural areas. The RA approach of interviewing key persons and not solely refugees that (mis)use substances allowed us to gather multi-perspective knowledge on this sensitive topic. Qualitative content analysis was applied, aiming at identifying determinants of substance (mis)use embedded in the post-migration context of refugees and understanding the underlying mechanisms.

**Results:**

One main result of the data suggests that the link between refugees’ countries of origin and their post-migration substance (mis)use is not as direct as often assumed. It is observed that refugees’ prospects and opportunities in receiving countries (e.g., work permits) undermine this commonly reproduced link. Further determinants are related to living conditions in German refugee shelters and social relations with peers and families. The influence of refugees’ living conditions can be summarized as potentially increasing substance availability and distress, whereas family separation produces a loss of control and responsibility, increasing the risk for substance (mis)use. Peers’ influence on substance (mis)use was reported to reflect a search for a sense of belonging.

**Conclusions:**

Given that refugees who (mis)use substances have limited to no control over the factors identified in our study to be associated with substance (mis)use, common treatment and prevention approaches are challenged. Furthermore, we recommend aiming for a holistic comprehension of refugees’ substance (mis)use by expanding the focus beyond individuals to the social ecological context in any attempt, including prevention, treatment, research, and policy.

**Supplementary Information:**

The online version contains supplementary material available at 10.1186/s13031-023-00499-9.

## Background

The prevalence of increased substance use and substance use disorder (SUD) as a consequence of war and (armed) conflict has become evident in previous research [1–4]. According to Greene et al. [5], this is due not only to SUD co-occurrence with exposure to traumatic events, distress, and general mental health problems but also to increased drug availability as a result of the “breakdown of social norms around substance use” (p. 17) or the failure of government control [4].

In addition to effects on substance use, conflicts and crises trigger migration. Humans in unbearable situations leave their homes, willing to move away in search of safety. Due to coercion, force, or compulsion triggering the migration process, it must be acknowledged as forced migration [6]. Increasing continuously from year to year, in May 2022, the number of forcibly displaced people reached 100 million, which exceeds 1% of the global population [7].

Due to the increasing number of displaced people and their vulnerability to substance use and SUD, it seems important to examine substance use within displaced populations. However, evidence of increased substance use among displaced populations compared to non-displaced populations is weak [1, 8]. Horyniak et al. [2] concluded that the estimates of hazardous/harmful alcohol use are heterogeneous, ranging from 4 to 36%, alcohol dependence from < 1 to 42%, and for not further specified drug dependence from 1 to 20%. In this systematic review, the majority of studies examined prevalence estimates of substance use disorders among refugees and asylum seekers in high-income countries (USA, Central Europe). It seems evident to Horyniak et al. [2] that heterogeneity in prevalence estimates results from the heterogeneous contexts receiving countries provide to arriving individuals. It must be acknowledged that the receiving context is shaped by regionally varying substance availability, substance use patterns, and social habits [9, 10]. However, the post-migration realities of refugees,^1^ including asylum legislation, and living circumstances must be considered (cf. [12]). In research, it is too often mistakenly surmised that receiving contexts all over the world are homogeneous [13]. Considering the importance of country-specific contexts, this paper focuses on the contexts of urban and rural Germany and attempts to unravel the factors that might induce increased substance (mis)use.^2^ Accordingly, this paper aims to answer the following question: Is there an increased risk for post-migration substance (mis)use embedded in the German post-migration context, and what are the underlying mechanisms?

Post-migration stressors and their effects on mental health have been acknowledged in holistic approaches of psychosocial [17] or public mental health perspectives [18, 19], even if their effects have been underestimated for a long time. For instance, within the ecological model of refugee distress, Miller and Rasmussen [20] shifted the emphasis on examining mental health issues among forcibly displaced populations away from pre-migration experiences to the post-migration social ecology of refugees. Social ecology refers to factors at multiple levels that shape the setting of everyday life. A systematic review by Li et al. [21] emphasizes the predicting value of post-migration stressors and their complex interplay on refugees’ mental health. One specific factor among refugees’ social ecology is the process of obtaining a recognized residence permit. Its impact on psychological well-being was shown in the duration of asylum processes [22], visa insecurity (e.g., due to residence permits limited in time) [23, 24], and asylum-related detention [25, 26], significantly increasing the risk of psychiatric problems. In contrast, Chen et al. [27] found no negative impact of asylum process–related stressors on mental health. Nevertheless, they showed resettlement-related stressors, such as loneliness, economic issues, and discrimination, to be strong correlates for mental health outcomes. For the German context, in particular, a recently published systematic review scrutinizes factors embedded in the German post-migration setting harmful to refugees’ mental health [28]. Across the 13 studies included, the authors identified the following factors to be significantly related to refugees’ mental health outcome variables: asylum status, accommodation, occupation, family, language, integration, and discrimination.

Regarding substance (mise)use as a particular aspect of mental health, a small-scale survey among African refugees living in Australia exposed heavy alcohol consumption as a coping mechanism for migration-related stressors, such as boredom and frustration [29]. Nevertheless, in the field of substance (mis)use, little research has been conducted within a comprehensive social ecology framework. Most of the research aims to estimate the prevalence rates of substance use disorders among refugee populations and consequently, fails to contextualize substance (mis)use and address the impact of post-migration settings. Therefore, we argue that, due to the heterogeneity of the receiving contexts, developing an in-depth understanding of the post-migration determinants of substance (mis)use is a far more suitable approach. The present study aims to fill this research gap and develop an understanding of the links and underlying mechanisms between refugees’ social ecology determinants and substance (mis)using behaviors in Germany as an example of one receiving context. This approach allows us to derive measurements not only for behavior-oriented prevention of substance (mis)use but also for condition-oriented (thus, structural) prevention.

## Method

### Rapid assessment (RA) methodology

The implementation of the project was inspired by heterogeneous qualitative and iterative inquiries summarized under the label Rapid assessment and response (RAR) methodologies. The special features of RAR in general are to take advantage of any source of existing information, approximating the issue of interest from diverse perspectives, and not only assessing the subject but also generating a responsive intervention in a participatory manner. Due to the project’s focus on the assessment aspect rather than the response aspect, it seems more accurate for the research presented to refer to the RA methodology [30]. In the last few decades, RA methods have mostly been applied in low-income countries and humanitarian settings involving displaced communities [31–33]. In the last decade, a few studies deployed the methodology likewise in high-income settings [34, 35]. These methods have been proven to gather knowledge about sensitive topics, such as substance use or HIV, whose affected populations might be difficult to involve in research [36, 37]. This results from the approach of RA data collection to acquire knowledge about a community by interviewing key persons, regardless of whether they are members of the community of study interest. This advantage provides not only increased anonymity for the interviewees. Due to RA’s abundance of multi-sectoral perspectives, the use of multi-source data, and their ongoing triangulation, this method likewise enables comprehensive and in-depth examination of broad topics of research interest. Regarding substance (mis)use among refugees in Germany, a lack of willingness of refugees that (mis)use substances to participate in research on this topic was expected, for example, due to the fear of legal consequences (cf. deterrence theorizing, [38]). Such reasons have been hypothesized to likewise decrease refugees’ use of addiction care services [5, 39, 40]. Therefore, applying RA in this study project seemed reasonable to evaluate social ecology determinants for (mis)using substances embedded in the post-migration context among refugees living in Germany and to understand the underlying mechanisms.

### Design and procedure

The multi-site data collection was part of a five-year intervention study consortium (PREPARE), funded by the German Ministry of Education and Research. Eight RAs were conducted in the German study sites Hannover, Bremen, Leipzig, Frankfurt (Main), Cologne, Munich, Hamburg, and Berlin. Each study site included the city and adjacent rural districts, which were defined by the population density as a maximum of one-ninth of the corresponding city’s population density.

Following a broad literature review that included various sources (e.g., newspapers, conferences, annual reports of addiction care services, and scientific publications), local networks, and key persons at each study site, able to provide any kind of knowledge concerning substance (mis)use among refugees, were searched for. Key persons were defined as professionals of addiction or refugee aid services in regular contact with refugees, policymakers for health and social services, and law enforcement professionals. Refugees who (mis)used substances, their family members, and stakeholders within refugee communities were likewise considered to be key persons. Following the RA methodology, at each study site, semi-structured interviews (SSIs) and focus group discussions (FGDs) were conducted with key persons from May 2019 until September 2021.

SSIs were conducted in a face-to-face setting usually at the interviewees’ workplaces or public spaces (e.g., cafe) by eight trained bachelor and master psychology students (7 female, 1 male) following an interview guide. Interviews took mostly place in the German language, and only two interviews were facilitated by an interpreter (Farsi-German). The interviewers’ guide included the following main questions:

- Which substances are used by refugees? Please assign the substances mentioned to one or multiple subgroups of refugees (e.g., defined by characteristics such as age, origin, gender, accommodation, and legal status)?
- Which problems do you perceive as the three most dominant substance-related problems among each subgroup?
- Which are specific factors affecting the substance use of each subgroup(s), related to e.g., their situation in Germany, the situation in their country of origin, availability, and price of substances in Germany? Which function does substance use have in the context of each subgroup?

Key persons were asked to focus on refugees who arrived in Germany after 2015. Regarding credibility, the SSIs were audio-recorded, anonymized, and transcribed verbatim following a simple transcription manual [41]. Of the two SSIs conducted with the facilitation of an interpreter, only the parts in German were transcribed.

Insights, discrepancies, and voids arising from and between the SSIs at the respective study sites were presented and discussed with local professionals in FGDs, aligning with RA methodology. The FGDs took approximately 90–120 min. Regarding credibility, FGDs were audio-recorded and detailed minutes were taken by someone other than the moderators of the FGD. The diverse FGD participants did not only validate the preliminary findings resulting from the SSIs but synthesized different perspectives and opinions discursively within their discussions. This approach stands in the tradition of communicative validation [42] or member-checking principles [43] as tools to reduce researcher bias and potentially enhance the trustworthiness and intersubjectivity of qualitative research.

### Recruitment and respondents

The identified key persons were contacted and invited to participate in the RA. Furthermore, the snowballing technique was applied to contact additional key persons. A total of 108 SSIs were conducted with 41 key persons who stated they were professionals in any kind of addiction care services, 46 professionals in refugee aid services, and 18 local policymakers or representatives of law enforcement institutions. In addition, 13 key persons stated that they were in contact with refugee communities as individuals. Several interviewees were affiliated with more than one category. Among the 108 key persons interviewed, 10 brought up biographical references to their own experiences as a refugee. However, almost all of them got in touch with the project due to their role as professionals in addiction care or refugee aid services regardless of their individual flight or substance (mis)use biography. One-third of the SSIs conducted (34 SSIs) referred to expertise in rural areas. At every study site, recruitment of key persons in rural areas was more challenging, while less expertise was detected than in urban areas. See Table 1 for a summary of the SSI respondents.

**Table 1:** Overview of SSIs and FGDs, including information about the respondents/participants

	Total	Key persons’ backgrounds (multiple affiliations possible)				Expertise from		Self-reported ‘refugee’
		Professional in addiction care services	Professionals in refugee aid services	Local policymakers, representatives of law enforcement institutions	No further specified individual contact with refugees	Urban areas	Rural areas	
Semi-structured interviews	108	41	46	18	13	74	34	10
Focus group discussions	10	n.a				2	2	n.a
						+6 combined FGD		

Recruitment of the FGD participants followed the approach of involving participants from diverse work fields (e.g., addiction care, refugee assistance services, refugee shelter, persons specialized in working with women/LSBTTIQ*). For an overview, see Table 1. Ten FGDs were conducted. On average, seven key persons participated (min–max = 5–10). Key persons who had been interviewed in SSIs were also invited to participate in the FGD to provide further knowledge or discuss discrepant expertise with the group. However, not all FGD participants had been interviewed before. Six FGDs combined key persons from urban and rural areas. In Hamburg and Berlin, separate FGDs for participants from the city and the adjacent rural areas were implemented to investigate the differences between the rural and urban contexts. Additionally, in these two study sites, rural networks were the most accessible. Four FGDs took place in person, and six groups met online via Zoom software due to the Covid-19 pandemic.

### Ethics approval and consent to participate

This research was approved by the ethics committee of the Charité – Universitätsmedizin Berlin (EA2/203/19). The questions asked did not aim for self-reporting of the respondents’ own substance (mis)use but always addressed the key persons’ knowledge of substance (mis)use of refugee communities in Germany in general or of indicated subgroups (third-person perspective). Key persons participated voluntarily and, first, were thoroughly informed about the objectives and methods of the research, and, second, gave their informed consent to confidentiality, data storage, and processing by signature. When transcribing the SSIs and taking the minutes of the FGD any kind of personal or institution-related information was omitted. Minutes and transcripts were pseudonymized by a letter from A to H referring each to one study site, an additional “L” if the interview referred to expertise from a rural area and a serial number. Audio records were deleted immediately after transcription/minutes were taken.

### Analysis

Qualitative content analysis of the SSI transcripts and FGDs was applied due to its efficiency in structuring large amounts of qualitative data [44]. The analysis was conducted with MaxQDA version 2020 [45] by three young scientists with a professional psychology background (LH, PS, AM). An overview of the coding scheme is provided as Additional file 1. Before starting, a coding scheme structured by themes was deductively created based on the key questions of the SSIs/FGDs; which are the characteristics of refugees that (mis)use substances? (Theme 1), Which substances are being (mis)used? (Theme 2), which factors affect substance (mis)use? (Theme 3). Distinctions between rural and urban study sites were captured within a fourth theme. Subordinated to the themes, deductively derived categories were added to capture the different aspects of the respective theme. For example, “age”, “family status” and “country of origin” were some of the categories affiliated with the theme “characteristics of refugees that (mis)use substances”, whereas “motives of substance use”, “situation in Germany” and “substance use related differences between contexts of origin and receiving context” were some of the categories associated with the theme “factors affecting substance (mis)use)”.

The coding process then started with three randomly selected documents from the dataset which in total included 118 documents. In the analysis, SSI transcripts and FGD minutes were treated equally. Each of the three coders coded independently. Text segments were coded with the respective category code, or, in case the segment did not fit with any of the deductively derived categories but was related to a key question, it was coded with the superordinate theme code. Afterward, codings were compared among the three coders, and categories were discursively differentiated into inductively derived, more specific codes capturing the specifications of the category. Coded text segments were accordingly moved from the category to the subordinate code. To give an example; subordinate to the category “situation in Germany”, codes such as “rights and opportunities in Germany” and “long asylum procedures and uncertain perspectives” were created. Where necessary, code definitions were cherished within a code memo. Text segments coded with the superordinate theme code were screened and discussed between the coders. If new aspects of the theme emerged, a, in this case inductively derived category, was added to the coding scheme. Each adaption within the set of codes made the recoding of all documents necessary. To avoid numerous recoding loops, the set of codes was not changed after it seemed able to capture the data adequately. The final coding scheme included four themes, 13 categories, and 59 codes.

Due to a continuously enhanced coding agenda, rule-based coding among all coders was ensured, and data could be analyzed not only qualitatively but also in terms of quantifying frequencies of single categories as well as in terms of contingency between different categories. Formative and summative reliability checks [44] were implemented in permanent contact and ongoing discussions between the coders during the entire coding process. Accounting for reflexivity, the procedure of analysis and interpretation was continuously and discursively reflected among the members of the project team and researchers of different levels of seniority, professional background, and migration-related experiences within regular colloquium sessions (i.a., SP, IS, UK). This procedure can be classified as a way of peer debriefing [46], contrasting the ‘member check’-like FGDs with key persons from the field. Altogether, those procedures contribute to high levels of dialogical intersubjectivity in the analysis and interpretation of our study results [47].

## Results

The result section is structured in the following manner. First, the important role of refugees’ prospects and opportunities within the German receiving context in the link between countries of origin and post-migration substance (mis)use is presented. Then, the relation between living conditions and substance (mis)use is examined as a matter of substance availability, evolving distress in refugee shelters, and the socio-spatial features of the respective accommodations. This is followed by a third section on the relevance of social contact. Therein, we report on the observation that the family separation increases substance (mis)use. Furthermore, we describe how social belonging is negotiated by substance (mis)use among peers. Separate presentation of the findings from SSIs and FGDs was perceived as redundant, as the FGDs were considered as resembling the general sense of the SSIs in their entirety. Regardless, wherever the FGDs brought up further or divergent aspects, the emerging of the finding from the FGD is reported as such.

### Refugees’ prospects and opportunities shape the link between countries of origin and post-migration substance (mis)use

Substance (mis)use was mainly reported among male refugees younger than 30 years. In addition to age and gender, key persons defined subgroups that (mis)use substances frequently by country of origin or language area (e.g., Farsi speakers). Afghanistan, Iran, and Syria were the countries of origin mentioned the most. Interestingly, when talking about refugees from African countries, key persons often did not name the country but referred to the continent. Narrations relying on continents/countries of origin suggested them to determine the pattern of substance (mis)use and substances consumed (i.a., H_8, B_4, FGD_CL). In contrast, other key persons (i.a., A_7, CL_4, B_8) completely neglected such a direct link between substance (mis)use and country of origin and offered alternative explanations: “I think I would not so much limit it to nationalities, but rather to the context in which the people here move around” (F_4, Pos. 22).^3^

Within the SSIs and FGDs, this setting was specified by enduring asylum cases and uncertain perspectives (e.g., in terms of family reunification; i.a., A_2, EL_2, FGD_C), precarious accommodation (i.a., HL_2, E_4, FGD_C) as well as limited rights and opportunities regarding meaningful activities (e.g., work permit, participation in integration/language courses, i.a., AL_4, F_5, FGD_C) and health care (i.a., CL_6, E_8, H_2). Key persons reported the link between the setting of refugees’ everyday lives, and substance (mis)use equally for rural and urban areas as follows:

In the vast majority of cases, it is the people who are sitting at home, who are not allowed to do anything. Uhm and actually have no prospects anymore and are just waiting to see what happens and are also afraid about what happens next. (GL_3, Pos. 20)

Where there are few prospects, where there is a lot of despair, there is often a high level of substance use, and the harder the situation and the less prospects there are, the higher I would estimate the risk [for substance use]. (H_6, Pos. 19)

Especially for refugees who have little hope and few prospects, some key persons believed that what refugees are jeopardizing with substance (mis)use seems to weigh less compared to the advantages of the (mis)use (e.g., self-regulating effect): “So they know that it [substance use] is filthy, but it’s not filthier than the situation they’re in at all” (E_2, Pos. 98).

**Fig. 1:**
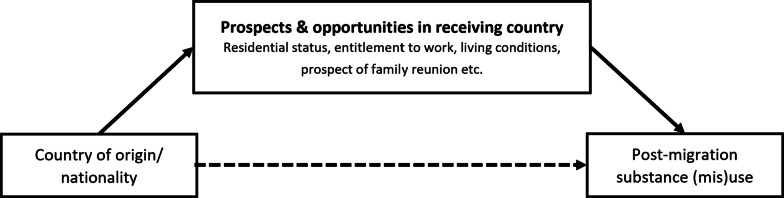
Link between refugees’ countries of origin and substance (mis)use in Germany shaped by the prospects and opportunities in the receiving country

The post-migration settings, prospects, and opportunities described are depending on the refugees’ countries of origin or their nationality.^4^ Thus, as shown in Fig. 1, our data suggests the link between countries of origin and substance (mis)use to be indirect and to a large extent shaped by refugees’ prospects and opportunities in the receiving country.

### The link between living conditions and substance (mis)use as a matter of substance availability, distress, and socio-spatial features

In general, refugees’ living conditions were dominant among the key persons’ reports on refugees’ substance (mis)use habits and the availability of certain drugs. The data conveys the impression that most of the refugees that (mis)use substances live in refugee shelters. In contrast, little was described about substance (mis)use among refugees who live in private spaces; potential confounding between the type of accommodation (shelter vs. private) and residential status, as described above (see Fig. 1), must be considered. The reasons for high substance (mis)use in refugee shelters surmised by the key persons (i.a., H_12, E_10, FGD_G), were in the first place related to the little privacy and autonomy entailed by life in a shelter:

Especially when I’m sitting in a facility like that for a long period of time, uhm, I am only allowed to cook at certain times, only allowed to take a shower at certain times, only allowed to do laundry at certain times, I don’t have anything to do all day, uhm, then there are factors that actually, uhm, eventually put pressure on the psyche, and can not only increase the use of addictive substances but actually also lead to a change in mood*.* (CL_2, Pos. 73)

But the other flatmates in this room [...], they want to [...] smoke, smoke pot, consume, listen to music and that is often a problem for them. They don’t want to be in this room anymore, and they want somehow, either somewhere else or a single room for themselves. But this is not available at all. (C_8, Pos. 69)

In addition, interviewees and FGD participants reported the remarkable availability of substances in refugee shelters due to drug dealing (i.a., A_4, C_8, GL_2), as well as fellow housemates recommending substance use based on their own “positive” experiences, for example, to tackle insomnia, without explaining or knowing what the substance is and what risks it brings (i.a., C_3).

I think that if you don’t use drugs now and you share a room with someone who does use drugs, it can either put you off or maybe lead to you being infected. So, I think that it has more to do with the social context*.* (F_4, Pos. 22)

And the initial contact worked [...] via people from one’s own culture, one’s own language, who then said, ‘You’re so sad, you’re under so much stress. Have a smoke!’ And some had an idea about what they were consuming, and others did not*.* (A_4, Pos. 21)

When discussing unaccompanied minors, who in Germany are usually housed in youth welfare living facilities, some interviewees identified these closely supportive living contexts as a protective factor because strict rules are applied; in contrast, community shelters were associated with far less support, supervision and rules for their residents (i.a., G_8, H_5, EL_2). Accordingly, several key persons (i.a., B_7, FGD_A, FGD_G) pointed to the age-related obligation to move into bigger community shelters, as a critical moment regarding the personal development of young adults and substance (mis)use:

And I think it’s difficult to make the transition at all from an unaccompanied minors facility, which is very supportive and very intensive and has surely somewhat replaced the family. Most of them were simply kicked out without mercy as soon as they turned 18 [...] And that means they moved into the shared accommodation on their 18^th^ birthday. And that was not a good transition*.* (E_7, Pos. 66)

In contrast, if teenagers were believed to have succeeded in creating meaningful future perspectives, they seemed to easily quit (mis)using substances (i.a., E_2, CL_5, HL_2). This reinforces the expounded relation between refugees’ social ecology and substance (mis)use:

So if they have prospects and a path that they can follow, then smoking pot no longer plays any role at all, for 2/3 of those who did it before. So, it really drops rapidly then and, uhm, is also stopped by itself*.* (C_4, Pos. 125)

In addition to the examination of refugee shelter facilities as social ecology settings themselves, the data shows that the socio-spatial locating of refugee shelters within rural areas and likewise within cities were reported to affect refugees’ substance (mis)use. Especially in rural areas, the social ecology component was emphasized for refugee shelters located, for example, “in the middle of the forest without Internet” (CL_5, Pos. 92) or in areas where “at night, [it] is really very dark here already on [the] street” (CL_5, Pos. 124). Few activities, restricted mobility, and limited autonomy were believed to increase boredom among refugees located in rural areas and thus increase substance (mis)use (i.a., CL_4, GL_5, FGD_C). These links were reported, although illicit drugs were perceived as less available in rural than in urban areas (i.a., AL_5, FGD_E):

Community accommodations […], are mostly in a relatively rural area and not so well connected. Which is also often a problem. Then they have to somehow; then there is only one bus then and then. (...) The less self-determined one lives, the more one consumes, as one can imagine, that it is simply a stress factor*.* (HL_4, Pos. 17)

An issue frequently raised by the interviewees and taken up by FGD participants was boredom (i.a., CL_5, AL_4, FGD_F). Taken together with key persons’ assumption that the deficient and poor accessibility of (mental) healthcare, addiction care, or prevention services in rural areas (i.a., GL_5, HL_1, FGD_AL), those factors were described as relevant, especially for refugees with little German language proficiency; they believed substance (mis)use to be hereby encouraged or maintained among refugees in rural areas. In addition, higher availability of substances, a sense of belonging to a community (e.g., the urban open drug scene), and health care services were described as pulling refugees either occasionally or permanently from rural to urban areas (i.a., E_7, FGD_A, FGD_CL). According to the data, refugees were surmised to do so although they would risk losing their right to accommodation, government benefits, and/or legal consequences because residential status comes sometimes with restricted freedom of movement (e.g., restricted to one administrative district; i.a., HL_2, FGD_A, FGD_CL).

Moreover, in urban study sites, the issue of the location of a shelter was likewise perceived as crucial and potentially affecting substance (mis)use. For instance, at one study site, several interviewees (i.a., E_4, E_7, FGD_E) reported a significant number of refugees that (mis)use substances who had arrived as unaccompanied minors and were at the time accommodated in a hostel near to the open drug scene.

### Absence of refugees’ families and social belonging influence refugees’ substance (mis)use

Social contacts or their absence were observed to affect refugees’ post-migration substance (mis)use, regardless of whether the key persons were talking about refugees in rural or urban areas. For instance, a large share of refugees that (mis)use substances was described as being in Germany without their families (i.e., children, partners, parents), just like the unaccompanied minors mentioned above. This seems to be the case as well for women; for instance, women traveling solo were reported to (mis)use alcohol, cannabis, and illicit drugs (i.a., GL_3, FGD_G, FGD_CL), whereas women with children and partners mainly stand out due to non-medical use of pharmaceuticals (i.e. B_4, EL_3, FGD_AL). The former were assumed to be older than in their mid-20s, including several transwomen.

The underlying mechanism of solo traveling as a determinant of substance (mis)use was described as having two parts. On one hand, substance (mis)use was reported as a consequence of the loss of structure, responsibility, and *“social control”* (G_2, Pos. 20), previously imposed by families (i.a., C_2, DL_4, FGD_E). In other words, as described by a refugee interviewee: “Those who don’t have any family at all, attachment and control go missing” (A_6, Pos. 4). On the other hand, key persons associated the absence of refugees’ families with experienced loneliness, missing sorely the family members left behind and worrying about their lives (i.a., A_6, C_8, HL_1).

In addition to conditions provided by having migrated with or without family, regarding peers and how they are associated with substance (mis)use, teenage refugees stand out in our data. Interviewees reported frequently an affiliation with the age-related peer trend of (mis)using cannabis and alcohol. Several interviewees (i.a., B_6, F_8) perceived these behaviors as offering teenagers a sense of belonging:

I think it’s also because, in the usual contexts, such as school, they were also integrated here [...], and then they did what the others were doing (laughs). So, you could also see it a bit as, well, integration in the traditional sense. They also did what was typically available here at this point*.* (EL_2, p. 28)

Amid forced migration, belonging to any social group in the receiving context seems to be a relevant motive for (mis)using substances (i.a., A_3, H_2, GL_4). To give another example from the SSIs; communities built up over years in Germany (e.g., the Iranian community) were described as offering not only peer contact and a sense of belonging for refugees who had arrived recently, but also increase the availability of substances that one might not expect to be widespread in the receiving country (e.g., opium; i.a., A_5, E_2, E_11).

In contrast, regarding ecstasy pills as an illicit drug widely used by young people in Germany, there are few reports by key persons on ecstasy (mis)use by young refugees. One reason prominent in our data is the inadequacy of psychostimulants for refugees’ motives for substance (mis)use. Additionally, during an FGD, participants discussed a divergent assumption related to the lack of accessibility of nightlife venues for refugees due to discrimination and the thus decreased influence of peer trends related to substance (mis)use existing in those social contexts: “It is difficult for young refugees to access party drugs due to discrimination at the doors of clubs and pubs and high prices” (FGD_G, Pos. 32).

## Discussion

Altogether, 108 semi-structured key person interviews and 10 FGDs based on a multi-site rapid assessment were analyzed, aiming at identifying determinants of substance (mis)use embedded in the post-migration context of refugees and understanding their links and underlying mechanisms. As one main result, the link between refugees’ countries of origin and their post-migration substance (mis)use is suggested to be not as direct as often assumed. It appears that refugees’ prospects and opportunities in receiving countries undermine this commonly reproduced link. For instance, the work permit, prospects for family reunions or permanent residency as well as the individual’s residential status depend on the country of origin and thus significantly shape the setting of refugees’ everyday lives. Further determinants of special relevance within the substance (mis)use affecting social ecology are related to living conditions and social relations with peers and families. The role of the former can be summarized as potentially increasing substance availability and distress. Additionally, the data substantiates an interplay between refugees’ (lack of a) sense of belonging and substance (mis)use.

In contrast to dominant explanatory approaches to substance (mis)use focusing on the period before migration or individual psychological conditions, the social ecological approach applied to the data acknowledges substance (mis)use as maintained and facilitated by structural factors in the receiving country. A recently published systematic review of qualitative research [40] supports our findings on the influence of peers and family separation on refugees’ substance (mis)use. In addition, the authors mention the challenge of integration and the lack of education and employment as core motives for refugees (mis)using substances. Whereas they do not refer to the influence of different types of accommodation, they emphasize the risk due to the high availability of substances, for instance in Germany [50]. The negative effect of refugee shelters on substance (mis)use has been reported but the data is inconclusive about the underlying mechanism [2]. Our data suggests that it is not only the general distress and restricted autonomy imposed by life in a refugee shelter, that generally harms refugees’ mental health [51–53] and fosters refugees’ substance (mis)use, but also the fact that in those shelters availability of substances is particularly high. As quantitative data investigating the substance (mis)use among refugees in Germany from a social ecological stance to our knowledge, does not yet exist, our findings cannot be supplemented with quantitative studies. Nonetheless, a large number of quantitative studies identified similar factors as our study when examining post-migration stressors’ impacts on the mental health of refugees in Germany in general [28] and qualitative approaches have suggested mechanism similar to the ones suggested by our data [53].

Our findings challenge common substance misuse prevention and treatment approaches. Just as in research, their focus used to be on behavioral and individual factors of substance (mis)use. In the case of refugees and migrants, for decades, the obstacles to accessing mental healthcare services were attributed mainly to those individuals (e.g., lack of information) or their culture [54]. Therefore, culturally grounded prevention and treatment programs for specific communities were suggested [55–57]. These approaches have in common a deficit-oriented perspective of refugees that has been criticized for years and that ignores the surrounding context conditions. Criticism of the decontextualization of social problems, previously frequently voiced in social science, is also applicable here [58–60].

Based on the findings of this study, in the first place, we first and foremost recommend substance (mis)use prevention measures to expand from behavioral to structural prevention by fundamentally changing the social ecology of refugees for the better. Therefore, the translation of the findings into policy recommendations is relevant. For example, the finding that solo travelers are at elevated risk of substance (mis)use is not particularly new [24, 40, 50]. From a strand of literature, we know about the buffering effect of family support on substance use [61–63]. For Germany in particular, a longitudinal study revealed family reunifications, with nuclear family members or siblings, to positively affect generally refugees’ mental health [64]. Therefore, in the examination of refugees’ social ecology, legal obstacles regarding family reunification must be scrutinized.

In a similar vein, we discussed the data substantiating how the sense of belonging to a community is offered or negotiated by substance (mis)use. This goes beyond the mere description of drinking alcohol to socialize with peers [29], as it addresses categories of identity, social belonging, inclusion, and exclusion, which especially when examining migrants/refugees, seem to play a significant role. For instance, the systematic review by Hajak et al. [28] encompasses studies pointing out loneliness and experiences of discrimination to be strongly associated with poorer mental health. Accordingly, in the words of Lindert et al. [50] we conclude, that substance (mis)use depicts an “active coping behavior to increase acceptance and belonging to the host country” (p. 22). It might be redundant to point out the maladaptive potential of such guideways to integration. In addition, it must be acknowledged that refugees that (mis)use substances face barriers when aiming for equal participation in society on two levels, being a refugee and being a substance (mis)user.

Another realm in need of policy changes identified by our study is refugee accommodation. This demand is not novel, as the distress entailed by living in a refugee shelter had been described extensively [51–53] and advantages of private and decentralized housing compared to refugee shelters on the psychological well-being of refugees particularly in Germany have been shown to be significant [28]. Our study supports the need for decentralized housing as it additionally identifies shelter accommodation as a risk of substance (mis)use. Thus, our findings point out experiences of social exclusion due to being accommodated in shelters in the socio-spatial periphery, in addition to the aforementioned influence of distress, limited autonomy, and high substance availability entailed by living in a refugee shelter. Those experiences were believed to increase boredom and in turn substance (mis)use. Similar links were described for refugees’ mental health in general [53, 65]. It must be considered that once a certain affinity for substance (mis)use is developed, for example, as a coping mechanism for psychosocial distress, the risk of SUD is elevated. For instance, a recommendation underpinned by empirical findings, and informed by theory, is to prevent or quit substance (mis)use by changing social networks [66–68]. However, in practice, even when refugees are willing to change their accommodations, they may face barriers, or in the worst case be forced to stay in a social environment shaped by high substance availability. Therefore, another relevant structural prevention measure, not only, but also in terms of substance (mis)use, is to strengthen refugees’ autonomy in terms of housing options as soon as possible after their arrival.

In the light of the link between limited prospects and opportunities for refugees and their post-migration substance (mis)use, the issue of boredom and restricted access to labor stood out. Interestingly, within the field of substance misuse treatment and (relapse) prevention, self-efficacy beliefs [69, 70], employment [68, 71, 72] and any other meaningful activity [73] alternative to using drugs have been generally discussed as key issues. This is opposing the fact that refugees have limited to no control over related domains e.g., long-term prospects in receiving countries, work permits, accommodation, and family reunions. This lack of control itself has been shown to harm refugees’ psychosocial well-being and mental health [52]. The need for quick clarification of the refugees’ prospects in Germany has been emphasized, as it appears to facilitate structural integration [53, 74].

In summary, changing refugees’ social ecology for the good, aiming to offer them an opportunity to participate effectively and equally in receiving societies, seems important in terms of structural substance (mis)use prevention. Regardless of substance use, offering refugees a sense of belonging to the receiving society must be considered a macrosocial responsibility [59].

In addition to the afore-mentioned points addressing structural prevention measurements, the data is equally of value to inform behavioral prevention and treatment implications for refuges that (mis)use substances. First, the rich findings related to the influence of peers on substance (mis)use improve the planning of information-dissemination approaches as prevention measures and might inform community-based interventions [75, 76] by providing an understanding of different refugee communities and their dynamics. Second, in treatment settings, we encourage practitioners to not only focus on individuals’ pre-migration experiences or assumptions attributed to their countries of origin but instead take a holistic stance and examine the multiple factors within the post-migration setting and evaluate any possibility to improve it.

## Limitations and strengths

The RA conducted offers broad insight into substance (mis)use among refugees living in Germany based on diverse perspectives. Nonetheless, the third-person characteristic of interviewing key persons, regardless of their belonging to the target community, limits the data; only a minority of respondents and FGD participants drew biographic references to their own experiences as refugees and/or substance (mis)use. Even though recruitment activities aimed for the inclusion of key persons affected by their own flight and substance (mis)use experiences, and language interpreters to conduct SSIs in any other language than German were easily available, the assumption that people and in particular refugees that (mis)use substances, are hard to reach for research has been confirmed. We need an understanding of how refugees’ participation in studies on substance (mis)use might be encouraged (e.g., incentives and anonymity).

Although interviewing key persons not solely of clinical backgrounds offers a more holistic view of the social ecology of refugees, it brings with it the limitation of a non-uniform use of the terms describing the severity of substance (mis)using behaviors. This made it impossible to distinguish between substance use, misuse, addiction, or any other type of SUD within the analysis. If aiming at differentiation between forms of substance (mis)use, a study must be related to a clinical diagnosis while thoroughly reflecting on the issue of cultural sensitivity of respective screening instruments [77].

In addition, our data did not allow for contrasting key persons from diverse professional backgrounds or key persons affected by (forced) migration and/or substance (mis)use themselves with other key persons. Nevertheless, the vast number of semi-structured interviews conducted and the implementation of FGD as a communicative validation of preliminary results strengthened the validity of the study.

Although this study was limited to Germany and findings are not necessarily transferable to other countries, we still tried to account for the common but incorrect assumption that post-migration settings would be homogenous [13], by using a multi-site design and comprehensive involvement of urban as well as rural areas. Nonetheless, the holistic nature of the data, offering insights into the mechanisms underlying the risk of increased substance (mis)use embedded in the social ecology of refugees in Germany, might contribute to conceptual frameworks. This should be acknowledged as a core advantage of empirical qualitative research [40].

## Future research

To our knowledge, this study is one of the first to address determinants of substance (mis)use embedded in a refugees’ post-migration social ecology perspective. Although within the last few years growing evidence for social determinants of mental health has been extended to refugee populations (cf. [65, 78]), this has rarely occurred in the field of substance (mis)use or SUD. We encourage researchers to examine the bigger picture by expanding the focus to the social ecological context. Future research could extend the focus on the role of pre- and post-migration social norms and attitudes toward specific substances or substance (mis)use and investigate their influence on post-migration substance (mis)use. Furthermore, the qualitative exploration in this article might be tested within a sound quantitative survey for statistical significance. In addition, future research could investigate different SUD based on diagnostic criteria while accounting for the cultural and social sensitivity of terminologies related to substance (mis)use [15, 16] and related challenges emerging when using screening instruments [77]. However, such an approach makes it necessary to directly study refugees that (mis)use substances. Therefore, it must be understood, why refugees might be reluctant to talk about their own substance (mis)using behaviors to researchers and how those concerns might be overcome. Possibly, contrasting the perspectives of e.g., key persons from addiction care and refugee aid services, might offer relevant insights and may inform interventions on how to make addiction care services more accessible to refugees.

## Conclusion

The analysis of integrated data from the multi-site qualitative RA conducted highlights the relevance of examining the multi-level factors shaping the setting of refugees’ everyday lives when aiming at investigating substance (mis)use among refugees. The data allow us to concretize refugees’ social ecology, as displayed in the model of refugee distress [20]. Factors identified as crucially related to substance (mis)use include refugees’ post-migration prospects and opportunities, accommodation, family separation, and a general wish for a sense of belonging. Given that those factors predominantly underlie integration policy frameworks, legal restrictions (e.g., on family reunions, accommodation, and work permits) should be reconsidered in light of their negative impact on mental health and substance (mis)use and related treatment costs. Moreover, general attempts applied in prevention and treatment, such as alternative activities to drugs, seem to be only slightly applicable to refugee populations because refugees have limited to no control over domains such as work permits and living environments. Therefore, we strongly recommend aiming for a holistic comprehension of refugees’ substance (mis)use by expanding the focus beyond individuals toward the social ecological context in any attempt, including prevention, treatment, research, and policy.

## Supplementary Information

**Additional file 1**: Coding scheme applied to the Semi-structured interview transcripts and minutes of focus group discussions.

## Data Availability

Due to the persisting sensitivity of the data despite de-identification procedures as well as the lack of consent for sharing the original interview data, the transcripts of interviews and notes of group discussions cannot be made publicly available.

## Abbreviations

FGD: Focus group discussion
SSI: Semi-structured interview
RA: Rapid assessment
RAR: Rapid assessment and response
SUD: Substance use disorder
ICD: International Statistical Classification of Diseases and Related Health Problems

## Footnotes

1 The term refugees used throughout the study and the article refers to the common parlance definition of the term, not a legal definition; refugees comprise different groups of forcibly displaces populations, including forced migrants, undocumented migrants, asylum seekers, and recognized refugees as they share the commonality of ‘perceived forcedness’ to emigrate [11].

2 Using the term '(mis)use' in the reporting of our study, we refer to any kind of substance use, whether it is recreational, excessive, substance misuse, substance abuse, or qualifying for substance dependence and regardless of the type of substance (i.a., legal, illegal, pharmaceuticals) (c.f. [14].Thereby, we acknowledge, that empirical evidence underpins the risk of misinterpretation and misunderstandings when relying on the universal character of medical terminology to describe substance (mis)use, due to the socially and culturally informed nature of such terminology concepts [15, 16].

3 Original quotes from SSIs and FGDs are in German. As for credibility, they were translated into English by one person and then translated back to German by a second person; both persons were not affiliated with the project. The main researcher compared the original and translated German versions of the quotes and adapted the English translation where necessary.

4 Refugees’ social ecology is determined legally by their residential status. Interestingly, in Germany, the latter is significantly dependent on the asylum seeker’s country of origin (e.g., due to reports on the national status related to asylum and deportation issued by the Federal Foreign Office [48] or bans on deportation related to the country of origin [49]).

## References

[1] Ezard N (2012). Substance use among populations displaced by conflict: a literature review. Disasters.

[2] Horyniak D, Melo J, Farrell R, Ojeda VD, Strathdee SA (2016). Prevalence and risk factors for substance use among refugees, internally displaced people and asylum seekers: findings from a global systematic review. Ann Glob Health.

[3] Jack H, Masterson AR, Khoshnood K (2014). Violent conflict and opiate use in low and middle-income countries: a systematic review. Int J Drug Policy.

[4] Odenwald M, Hinkel H, Schauer E, Neuner F, Schauer M, Elbert TR (2007). The consumption of khat and other drugs in Somali combatants: a cross-sectional study. PLoS Med.

[5] Greene MC, Haddad S, Busse A, Ezard N, Ventevogel P, Demis L (2021). Priorities for addressing substance use disorder in humanitarian settings. Confl Health.

[6] IOM (International Organisation for Migration). Glossary on migration. Geneva, Switzerland: IOM; 2019. https://publications.iom.int/books/international-migration-law-ndeg34-glossary-migration. Accessed 20 Jul 2022.

[7] United Nations High Commissioner for Refugees (UNHCR). 100 million people forcibly displaced. UNHCR Refugee Statistics. https://www.unhcr.org/refugee-statistics/insights/explainers/100-million-forcibly-displaced.html. Accessed 20 Jul 2022.

[8] Weaver H, Roberts B (2010). Drinking and displacement: a systematic review of the influence of forced displacement on harmful alcohol use. Subst Use Misuse.

[9] Degenhardt L, Hall W (2012). Extent of illicit drug use and dependence, and their contribution to the global burden of disease. Lancet.

[10] Manthey J, Shield KD, Rylett M, Hasan OSM, Probst C, Rehm J (2019). Global alcohol exposure between 1990 and 2017 and forecasts until 2030: a modelling study. Lancet.

[11] Echterhoff G, Hellmann JH, Back MD, Kärtner J, Morina N, Hertel G (2020). Psychological antecedents of refugee integration (PARI). Perspect Psychol Sci.

[12] Thöle A-M, Penka S, Brähler E, Heinz A, Kluge U (2017). Psychotherapeutische Versorgung von Geflüchteten aus der Sicht niedergelassener Psychotherapeuten in Deutschland. Z Psychiatr Psychol Psychother.

[13] Salas-Wright CP, Schwartz SJ (2019). The study and prevention of alcohol and other drug misuse among migrants: toward a transnational theory of cultural stress. Int J Ment Health Addict.

[14] Pillet-Shore D (2021). Peer conversation about substance (mis)use. Sociol Health Illn.

[15] Penka S, Heimann H, Heinz A, Schouler-Ocak MJ (2008). Explanatory models of addictive behaviour among native German, Russian–German, and Turkish youth. Eur Psychiatry.

[16] Mirza MQ, Harrison EA, Chang HC, Salo CD, Birman D (2018). Community perspectives on substance use among Bhutanese and Iraqi refugees resettled in the United States. J Prev Interv Community.

[17] Interagency Standing Committee (IASC). IASC Guidelines on the Mental Health and Psychosocial Support in Emergency Settings. 2007. http://www.who.int/mental_health/emergencies/guidelines_iasc_mental_health_psychosocial_june_2007.pdf. Accessed 20 Jul 2022. 10.1080/09540261.2022.2147420 36502397

[18] Heinz A, Zhao X, Liu S (2020). Implications of the association of social exclusion with mental health. JAMA Psychiat.

[19] de Jong J (2002). Trauma, war, and violence: public mental health in socio-cultural context.

[20] Miller KE, Rasmussen A (2017). The mental health of civilians displaced by armed conflict: an ecological model of refugee distress. Epidemiol Psychiatr Sci.

[21] Li SS, Liddell BJ, Nickerson A (2016). The relationship between post-migration stress and psychological disorders in refugees and asylum seekers. Curr Psychiatry Rep.

[22] Laban CJ, Komproe IH, Gernaat HBPE, de Jong JTVM (2008). The impact of a long asylum procedure on quality of life, disability and physical health in Iraqi asylum seekers in the Netherlands. Soc Psychiatry Psychiatr Epidemiol.

[23] Nickerson A, Byrow Y, O’Donnell M, Mau V, McMahon T, Pajak R (2019). The association between visa insecurity and mental health, disability and social engagement in refugees living in Australia. Eur J Psychotraumatol.

[24] Bogic M, Ajdukovic D, Bremner S, Franciskovic T, Galeazzi GM, Kucukalic A (2012). Factors associated with mental disorders in long-settled war refugees: refugees from the former Yugoslavia in Germany, Italy and the UK. Br J Psychiatry.

[25] Filges T, Montgomery E, Kastrup M (2018). The impact of detention on the health of asylum seekers: a systematic review. Res Soc Work Pract.

[26] Steel Z, Silove D, Brooks R, Momartin S, Alzuhairi B, Susljik I (2006). Impact of immigration detention and temporary protection on the mental health of refugees. Br J Psychiatry.

[27] Chen W, Hall BJ, Ling L, Renzaho AM (2017). Pre-migration and post-migration factors associated with mental health in humanitarian migrants in Australia and the moderation effect of post-migration stressors: findings from the first wave data of the BNLA cohort study. Lancet Psychiatry.

[28] Hajak VL, Sardana S, Verdeli H, Grimm S (2021). A systematic review of factors affecting mental health and well-being of asylum seekers and refugees in Germany. Front Psychiatry.

[29] Horyniak D, Higgs P, Cogger S, Dietze P, Bofu T (2015). Heavy alcohol consumption among marginalised African refugee young people in Melbourne, Australia: motivations for drinking, experiences of alcohol-related problems and strategies for managing drinking. Ethn Health.

[30] Fitch C, Stimson GV, Rhodes T, Poznyak V (2004). Rapid assessment: an international review of diffusion, practice and outcomes in the substance use field. Soc Sci Med.

[31] World Health Organisation (WHO) and others. Rapid assessment of alcohol and other substance use in conflict-affected and displaced populations: A field guide. Geneva: WHO; 2008.

[32] Ezard N, Oppenheimer E, Burton A, Schilperoord M, Macdonald D, Adelekan M, Sakarati A, Van Ommeren M (2011). Six rapid assessments of alcohol and other substance use in populations displaced by conflict. Confl Heal.

[33] Kumar MS, Mudaliar S, Thyagarajan SP, Kumar S, Selvanayagam A, Daniels D (2000). Rapid assessment and response to injecting drug use in Madras, south India. Int J Drug Policy.

[34] Dupont HB, Kaplan CD, Braam RV, Verbraeck HT, de Vries NK (2015). The application of the rapid assessment and response methodology for cannabis prevention research among youth in the Netherlands. Int J Drug Policy.

[35] Shrestha S, Stopka TJ, Hughto JM, Case P, Palacios WR, Reilly B, Green TC (2021). Prevalence and correlates of non-fatal overdose among people who use drugs: findings from rapid assessments in Massachusetts 2017–2019. Harm Reduct J.

[36] Stimson GV, Fitch C, Rhodes T (1998). Rapid assessment and response guide on injecting drug use: draft for field testing (IDU-RAR).

[37] World Health Organization (WHO). Rapid assessment and response adaptation guide on HIV and men who have sex with men. Geneva, Switzerland: World Health Organization; 2004. https://www.who.int/hiv/pub/prev_care/rar/en/. Accessed 20 Jul 2022.

[38] Salas-Wright CP, Vaughn MG, Goings TC (2017). Immigrants from Mexico experience serious behavioral and psychiatric problems at far lower rates than US-born Americans. Soc Psychiatry Psychiatr Epidemiol.

[39] Hunner-Kreisel C, Penka S, Krieg S, Heinz A (2001). Latente Ausschließung: migranten und drogenhilfe. Kriminol J.

[40] Saleh EA, Lazaridou FB, Klapprott F, Wazaify M, Heinz A, Kluge U (2022). A systematic review of qualitative research on substance use among refugees. Addiction.

[41] Praxisbuch interview, transkription & analyse, audiotranskription. Marburg: Dr. Dresing und Pehl GmbH; 2008.

[42] Mayring P (2021). Qualitative content analysis: a step-by-step guide.

[43] Birt L, Scott S, Cavers D, Campbell C, Walter F (2016). Member checking: a tool to enhance trustworthiness or merely a nod to validation?. Qual Health Res.

[44] Mayring P, Kiegelmann M (2002). Qualitative content analysis— research instrument or mode of interpretation?. The role of the researcher in qualitative psychology.

[45] VERBI Software (2019). MAXQDA 2020 [computer software].

[46] Lincoln YS, Guba EG (1985). Naturalistic inquiry.

[47] Bae S (2017). Intersubjectivity. Int Encycl Commun Res Methods.

[48] Federal Foreign Office Germany. Asylum law. https://www.auswaertiges-amt.de/en/visa-service/-/229968. Accessed 20 Jul 2022.

[49] Federal Office for Migration and Refugees Germany. National ban on deportation. https://www.bamf.de/EN/Themen/AsylFluechtlingsschutz/AblaufAsylverfahrens/Schutzformen/Abschiebeverbote/abschiebeverbote-node.html;jsessionid=D6811FE222B0DD196E5522621ECE9616.intranet242. Accessed 20 Jul 2022.

[50] Lindert J, Neuendorf U, Natan M, Schäfer I (2021). Escaping the past and living in the present: a qualitative exploration of substance use among Syrian male refugees in Germany. Confl Health.

[51] Mehran N, Abu Juma J, Lazaridou F, Foroutan N, Heinz A, Kluge U (2021). Spatiality of social stress experienced by refugee women in initial reception centers. J Int Migr Integr.

[52] Miller KE, Rasmussen A (2010). War exposure, daily stressors, and mental health in conflict and post-conflict settings: bridging the divide between trauma-focused and psychosocial frameworks. Soc Sci Med.

[53] Walther L, Rayes D, Amann J, Flick U, Ta TMT, Hahn E, Bajbouj M (2021). Mental Health and Integration: a qualitative study on the struggles of recently arrived refugees in Germany. Front Public Health.

[54] Penka S (2004). Migration und Sucht. Notwendigkeit einer “Interkulturellen Suchthilfe”?.

[55] Castro FG, Alarcón EH (2002). Integrating cultural variables into drug abuse prevention and treatment with racial/ethnic minorities. J Drug Issues.

[56] Bume AW (2016). Advances in substance abuse prevention and treatment interventions among racial, ethnic, and sexual minority populations. Alcohol Res.

[57] Jefee-Bahloul H, Bajbouj M, Alabdullah J, Hassan G, Barkil-Oteo A (2016). Mental health in Europe’s Syrian refugee crisis. Lancet Psychiatry.

[58] Krieger N (2021). Ecosocial theory, embodied truths, and the people’s health.

[59] Phillimore J (2021). Refugee-integration-opportunity structures: Shifting the focus from refugees to context. J Refug Stud.

[60] Rapp MA, Kluge U, Penka S, Vardar A, Aichberger MC, Mundt AP (2015). When local poverty is more important than your income: mental health in minorities in inner cities. World Psychiatry.

[61] Curtis-Boles H, Jenkins-Monroe V (2000). Substance abuse in African American women. J Black Psychol.

[62] Rowe CL, Liddle HA (2003). Substance abuse. J Marital Fam Ther.

[63] Trucco EM (2020). A review of psychosocial factors linked to adolescent substance use. Pharmacol Biochem Behav.

[64] Löbel LM, Jacobsen J (2021). Waiting for kin: a longitudinal study of family reunification and refugee mental health in Germany. J Ethn Migr Stud.

[65] Jannesari S, Hatch S, Prina M, Oram S (2020). Post-migration social-environmental factors associated with mental health problems among asylum seekers: a systematic review. J Immigr Minor Health.

[66] Flay BR, Petraitis J (1994). A new theory of health behavior with implications for preventive interventions. Subst Use Misuse.

[67] Valente TW, Gallaher P, Mouttapa M (2004). Using social networks to understand and prevent substance use: a transdisciplinary perspective. Subst Use Misuse.

[68] Westermeyer J (1989). Nontreatment factors affecting treatment outcome in substance abuse. Am J Drug Alcohol Abuse.

[69] Chavarria J, Stevens EB, Jason LA, Ferrari JR (2012). The effects of self-regulation and self-efficacy on substance use abstinence. Alcohol Treat Q.

[70] Kadden RM, Litt MD (2011). The role of self-efficacy in the treatment of substance use disorders. Addict Behav.

[71] Cebulla A, Smith N, Sutton L (2004). Returning to normality: substance users’ work histories and perceptions of work during and after recovery. Br J Soc Work.

[72] DeFulio A, Donlin WD, Wong CJ, Silverman K (2009). Employment-based abstinence reinforcement as a maintenance intervention for the treatment of cocaine dependence: a randomized controlled trial. Addiction.

[73] Swisher JD, Hu TW (1983). Alternatives to drug abuse: some are and some are not. NIDA Res Monogr.

[74] Kosyakova Y, Brenzel H (2020). The role of length of asylum procedure and legal status in the labour market integration of refugees in Germany. Soziale Welt.

[75] Botvin GJ (1990). Substance abuse prevention: theory, practice, and effectiveness. Crime Justice.

[76] Holder HD (2002). Prevention of alcohol and drug “abuse” problems at the community level: what research tells us. Subst Use Misuse.

[77] Marth S, Jakubauskiene M, Schäfer I, Lindert J (2021). Substance use instruments for refugees-systematic review. Eur J Public Health.

[78] Purgato M, Tol WA, Bass JK (2017). An ecological model for refugee mental health: implications for research. Epidemiol Psychiatr Sci.

