# Supplementary material for: Substance (mis)use among refugees as a matter of social ecology: insights into a multi-site rapid assessment in Germany
Source: Confl Health. 2023 Jan 19;17:1. doi: 10.1186/s13031-023-00499-9 (PMC9850330; doi:10.1186/s13031-023-00499-9)
Supplement: Supplementary file 1 — Additional file 1: Coding scheme applied to the Semi-structured interview transcripts and minutes of focus group discussions. [file 13031_2023_499_MOESM1_ESM.pdf]

Additional file 1: Coding Scheme applied to the Semi-structured interview transcripts and minutes of Focus group discussions

*Note: Blue shaded rows indicate themes and categories derived deductively*

| |
|---|
| **Theme 1: Characteristics of refugees (mis)using substances** |
| **1.1. Residential status & respective prospects** |
| secure |
| insecure |
| **1.2. Education** |
| Educational level high |
| Educational level low |
| **1.3. Family status** |
| Solo-travelling/without family in Germany |
| With family |
| **1.4. Age** |
| > 18 y/o |
| 18-30 y/o |
| 30-45 y/o |
| > 45 y/o |
| **1.5. Country of origin** |
| Afghanistan |
| Syria |
| Pakistan |
| Iran |
| Iraq |
| "Africa" |
| Maghreb |
| Eritrea & Somalia |
| West Africa |
| Former CIS countries |
| Others |
| **1.6. Gender** |
| Queer |
| Female |
| Male |
| **1.7. Accommodation** |
| homeless |
| Private home |
| Living in youth welfare living facility |
| Living in refugee shelter |
| **Theme 2: Which substances are (mis)used?** |
| Tobacco |
| Alcohol |
| Cannabis / Cannabinoids |
| Heroin |
| Opium |
| Opioids |
| Cocaine |
| (Meth-)Amphetamines |
| Medicines |
| Medicines, legal |
| Medicines. illegal |
| Others |
| polytox |
| **Theme 3: Factors affecting substance (mis)use** |
| **3.1. protective factors** |
| **3.2. Motives for substance use** |
| craving, SUD manifest |
| (self-)medication to cope with somatic pain |
| Experience of community and belonging/lack of belonging and family support |
| Boredom, lack of meaningful activities |
| Self-regulation/self-medication to cope with psychological distress |
| Fun, party, “check it out” |
| **3.3. (Mis)use in country of origin** |
| No (mis)use in country of origin |
| Societal norms/attitudes towards drugs and drug use in country of origin |
| **3.4. Situation in Germany** |
| Living conditions |
| Rights & opportunities in Germany |
| Long asylum procedures, uncertain perspectives |
| Excessive demands regarding situation in Germany |
| Disappointment regarding situation in Germany |
| **3.5. Substance use-related differences between context of origin and receiving context** |
| Lacking competencies and knowledge regarding risks etc. |
| More risky patterns of drug use due to low quality of drugs |
| Taking drugs because they are highly available in Germany |
| High availability + low prices in countries of origin |
| **3.6. Other factors** |
| Role models & role conflicts |
| **Theme 4: Differences between rural & urban areas** |
